# Increasing sika deer population density may change resource use by larval dung beetles

**DOI:** 10.1371/journal.pone.0226078

**Published:** 2019-12-05

**Authors:** Hayato Yama, Tomoko Naganuma, Kahoko Tochigi, Bruna Elisa Trentin, Rumiko Nakashita, Akino Inagaki, Shinsuke Koike

**Affiliations:** 1 Graduate School of Agriculture, Tokyo University of Agriculture and Technology, Fuchu, Japan; 2 United Graduate School of Agriculture, Tokyo University of Agriculture and Technology, Fuchu, Japan; 3 Department of Ecology, UNESP Sao Paulo State University, Botucatu, Sao Paulo, Brazil; 4 Forestry and Forest Products Research Institute, Tsukuba, Ibaraki, Japan; 5 Institute of Agriculture, Tokyo University of Agriculture and Technology, Fuchu, Japan; 6 Institute of Global Innovation Research, Tokyo University of Agriculture and Technology, Fuchu, Japan; CONICET - Universidad Nacional de Tucumán, ARGENTINA

## Abstract

Because animal feces contain organic matter and plant seeds, dung beetles (Scarabaeinae) are important for the circulation of materials and secondary seed dispersal through burying feces. Dung beetles are usually generalists and use the feces of various mammals. Additionally, the larval stages have access to feces from only one mammal species leaving them susceptible to changes in animal fauna and variations in animal populations. Here, we explain the effects of resource availability changes associated with sika deer (*Cervus nippon*) overabundance on dung beetle larvae feeding habits in Japan. *δ*^15^N values were notably higher in raccoon dog and badger dung than in that of other mammals. A dung beetle breeding experiment revealed that the *δ*^15^N values of dung beetle exoskeletons that had fed on deer feces during their larval stage were significantly lower than those of beetles that had fed on raccoon dog feces. The *δ*^15^N values of the adult exoskeleton were significantly lower in a deer high-density area than in a low-density area in large dung beetles only. It is possible that the high-quality feces, such as those of omnivores, preferred by the large beetles decrease in availability with an increase in deer dung; large beetles may therefore be unable to obtain sufficient high-quality feces and resort to using large amounts of low-quality deer feces. Small dung beetles may use the easily obtained feces that is in high abundance and they may also use deer feces more frequently with increases in deer density. These findings suggest that a larval resource shift associated with deer overabundance may affect ecosystem functions such as soil nutrient cycling and seed dispersal.

## Introduction

In terrestrial ecosystems, decomposers maintain the material cycle system (i.e., the detritus food chain) by decomposing and converting organic materials such as plants and animal carcasses into inorganic materials [1, 2, 3, 4]. Among organic materials, feces of wild animals, especially mammals, are highly nutritious and relatively large; therefore, decomposition of feces by decomposers is an important process in the material cycle, and decomposers are largely insects [5, 6]. Dung beetles (Coleoptera: Scarabaeidae) are major insect decomposers and decompose animal feces as food resources as both larvae and adults. Dung beetle tunneller types promote the material cycle in the soil and effectively modify the soil structure, because they carry feces into tunnels they have dug [7, 8, 9]. Additionally, tunnellers also act as secondary seed dispersers as plant seeds within feces are brought underground with the feces [10].

Typically, dung beetles have generalist diets, therefore resource shifts are easy. They depend on changes in the population density of individual animal species, and with these changes dung beetles may select higher quality feces (higher nitrogen, amino acid, and fatty acid contents) or more abundant feces [11, 12]. However, large dung beetles need the large feces produced by large mammals [13]. Adult dung beetles are highly mobile and identify the species responsible for dung by smell [14, 15, 16]. They generally prefer, and can select, feces that have high contents of water, volatile fatty acids, butyric acid, or indoles [14, 15, 16, 17, 18]. In contrast, until they metamorphose, larval dung beetles eat the feces in which they were laid as eggs, i.e. belonging to a single species of animal. Parent dung beetles choose high-quality feces as food for their larvae [19], but differences in feces type on which the larvae feed does not affect the diets of adult dung beetles [15]. The larval digestive system differs from that of adults and, unlike adults, larvae can feed on feces containing plant fragments. Therefore, larvae can feed on a wider range of animal feces, and it is possible that the amplitude of the larval resource niche is greater than that of adults at the species level [17]. Thus, the larval diet may be affected more readily than the adult diet by changes in fauna or faunal population density or food resource.

In recent years, there has been an overabundance of sika deer (*Cervus nippon*) in Japan, which has directly or indirectly impacted ecosystems because of overgrazing of several plant types [20, 21]. Such effects are also seen in dung beetles, as dung beetle food resources have increased with increases in sika deer populations, thus positively affecting their populations [22, 23]. However, overpopulation of sika deer reduces dung beetle diversity by reducing the understory and thus possibly drying out feces [24]. These effects vary with the type and body size of dung beetles. The biomass of small and dweller species increases with increasing deer density, whereas large and/or tunneller species are not related to deer density [25, 26].

The overabundance of sika deer affects other, sympatric, mammals. Understory reduction by the deer, the deposition of their dung, or both, increases the raccoon dog (*Nyctereutes procyonoides*) and badger (*Meles anakuma*) population densities by increasing the earthworm abundance, their main food resource. Conversely, rodents have declined in the understory, thereby negatively affecting predators such as red foxes (*Vulpes vulpes*) and Japanese martens (*Martes melampus*) [27, 28]. The diets of Asian black bears (*Ursus thibetanus*) have also changed, as they eat more deer meat [29]. Overabundance of sika deer thus may affect the relative amounts and compositions of feces excreted by each mammal species. Therefore, the diet of dung beetles may change because of changes in the density of sika deer populations.

Our objective here was to study the relationship between the increase in sika deer population density and the diet of dung beetles and changes in the dung beetle community. We hypothesized that dung beetle larvae would use more sika deer feces in areas with a high density of sika deer, because deer feces would be easier to obtain in these areas. We also hypothesized that this trend would be more notable in the larger dung beetle species, because large dung beetles need large amounts of feces. Because deer live in herds, we expected that increased size or abundance of these herds would make their feces easier for large dung beetles to find. If the larger dung beetles change their resource use to sika deer feces, then there would possibly be a change in ecosystem functioning. Specifically, larger dung beetles play a role in bioturbation through moving large quantities of earth to the soil surface during nesting, have a positive effect on permeability, and perform secondary seed burial for several plant seed sizes [6]. Because of the high sensitivity of dung beetles to habitat modification and changes in dung resources, many of these ecological processes may have already been disrupted [6]. Thus, clarifying the possibility of resource use change by dung beetles can explain the actual functional consequences of dung beetles.

To evaluate the dietary changes of larval dung beetles, we focused on the adult exoskeleton. Generally, insects that completely metamorphose form an exoskeleton, the molecular composition of which varies depending on food ingested by larvae [12]. Furthermore, we evaluated the larval diets of dung beetles by analyzing the nitrogen stable isotope ratios in adult dung beetles’ exoskeletons. Stable nitrogen isotope ratios exhibit stepwise enrichment through the food chain, with the values of *δ*^15^N increasing [30, 31]. Therefore, the values should differ notably between herbivores and carnivores [32]. Only a few studies have compared the values of *δ*^15^N in herbivore and carnivore feces or used *δ*^15^N to estimate dung beetle larval diets [12, 19]. We first used a breeding experiment to check whether differences in the stable nitrogen isotope values of larval food led to differences in the stable nitrogen isotope values of adult exoskeletons. Second, we collected different dung beetle species in two study areas where sika deer population densities differed; we then analyzed the nitrogen stable isotope ratios in the exoskeletons of the beetles and compared the values between the areas with different sika deer population densities to clarify the difference of the larval diets of dung beetles. Given the results, we discuss the reasons for differences in larval diet between areas and among different species of dung beetle.

## Materials and methods

### Study areas

The study was conducted in the Ashio–Nikko mountain area of central Japan (36.54–36.80°N, 139.22–139.49°E). We established 20 study sites, all in forested areas comprising deciduous trees, dominated by *Quercus crispula*, *Acer mono*, and *Carpinus japonica*. The sites were located at 850 to 1450 m above sea level, with an average altitude of 1120 m. Several medium to large mammalian species are found in the region, including sika deer, Asian black bear, Japanese macaque (*Macaca fuscata*), wild boar (*Sus scrofa*), badger, and raccoon dog.

**Fig 1:**
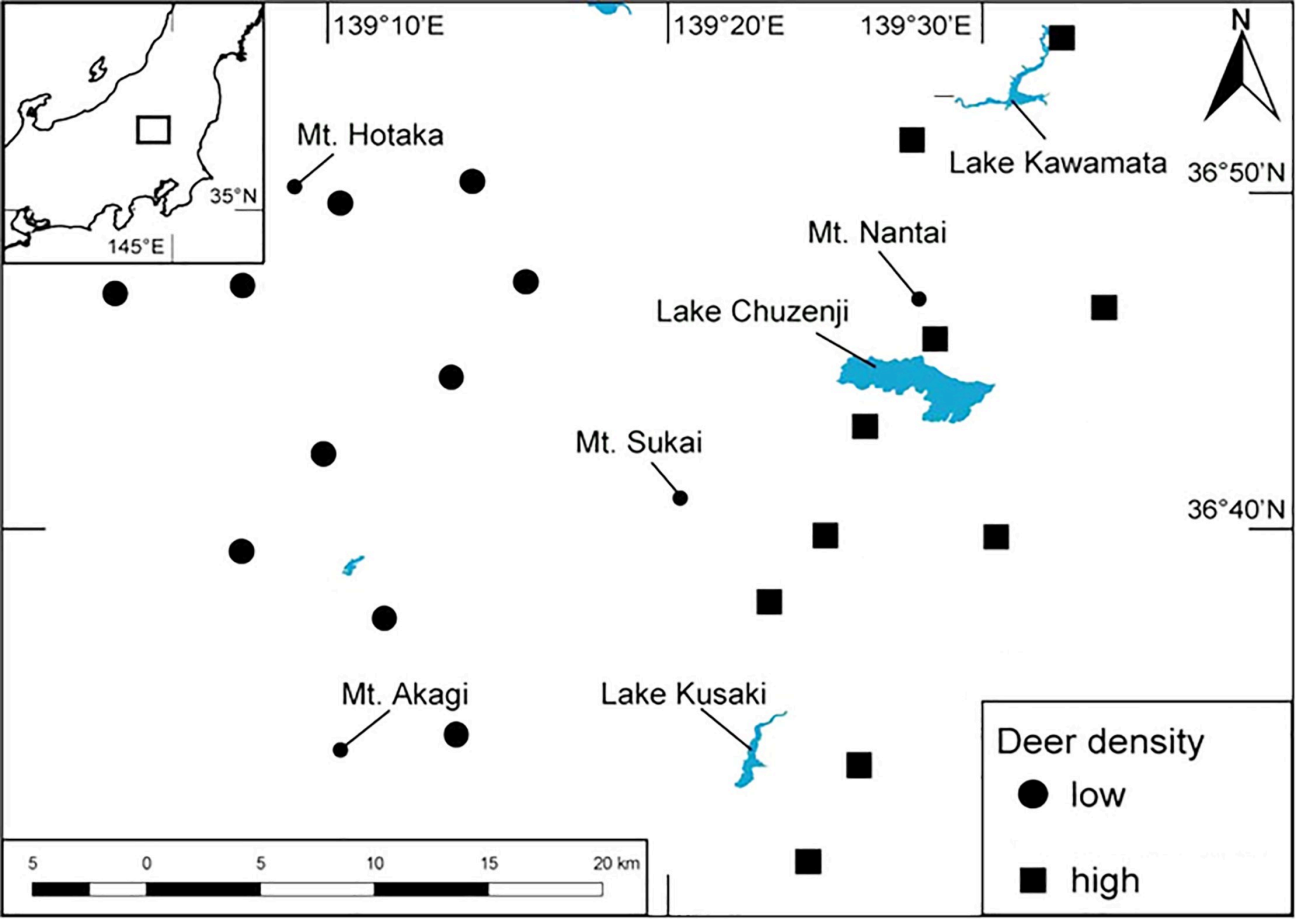
Location of the study sites.

The two study sites were split according to deer density: high (>15 deer/km^2^) (HD) and low (<5 deer/km^2^) (LD); 10 study sites were set up in each of the different sites of deer density, which were at least 5 km apart among sites and they were interspersed (Fig 1). Mountains more than 1500 m elevation occur among the two categories areas, and there are some mountains and valleys between each site. Deer densities in this region have been maintained at near constant levels for more than 10 years, as determined from data derived from previous reports [33, 34].

### Study design

Here, we first conducted a breeding experiment with dung beetles in which we compared the stable nitrogen isotope values of the exoskeletons of beetles that, as larvae, had fed exclusively on the feces of herbivores and of those that had fed only on the feces of carnivores. Second, we collected different dung beetle species and sampled the feces of each mammal species in the two areas with different sika deer population densities. We then analyzed the nitrogen stable isotope ratios in the exoskeletons and compared the values between the areas. Additionally, in each study area, we used an automatic camera to estimate the relative abundance of each mammal species to determine the effect of the availability of different types of dung on the dung beetles’ diet. By using the existing literature or data obtained on captive mammals, we then estimated the relative amount of feces of each mammal species present in each study area.

Because this study was performed in a national forest, we got permission from the Numata District Forest Office and the Nikko District Forest Office of the Forestry Agency to enter the national forest.

### Dung beetle breeding experiment

We bred three species of dung beetle: *Phelotrupes auratus*, *Caccobius nikkoensis*, and *Onthophagus lenzii* collected from the study area in late May 2017. These three species are all commonly found in the study area and are known to lay eggs during June and July [35]. Two males and two females from each collected species were kept in a container (a bucket with a depth of 50 cm and 30 cm diameter) filled with red soil to 40 cm depth (to allow for formation of tunnels for egg-laying). The container was covered with a cheesecloth to stop the dung beetles escaping, and animal feces (as feed) were placed on the soil surface. Animal feces were those of sika deer and raccoon dogs from a zoo (Tama Zoological Park, Tokyo). The captive sika deer mainly grazed on grass, whereas the raccoon dogs ate mainly customized sausage. This sausage was made especially for zoo animals and contained chicken, vitamins, and minerals. For each species, five containers for the addition of sika deer feces and five for the addition of raccoon dog feces were used. The dung beetles were placed in the container and 200 g of feces was added every five days. We left the feces until all females or all males had died, or two months had passed. Subsequently, we kept the soil moist and at an ambient temperature and observed the feces until October. On the emergence of new adult dung beetles, we recovered the beetles.

### Dung beetle fauna

We surveyed the dung beetle communities including both dweller and tunneller at each study site by setting one pitfall trap in each plot for three nights in June 2017, because a previous study had revealed a high frequency of dung beetles in early summer [36]. We established nine study plots within each study site; they were at least 300 m apart to minimize or eliminate the possibility of trap interference [37]. Plastic containers (77 mm diameter, 96 mm depth) containing saltwater as a preservative solution were buried to the rim in the ground, and deer dung placed inside a plastic mesh bag was suspended over the top. The results of our investigation of the dung beetle community would therefore be biased toward beetles attracted to Sika deer dung. However, a previous study of the dung beetle fauna (eight species) in this area [25] showed that these species of adults opportunistically use the feces of several species of mammal, including Sika deer [35]. Additionally, in central Japan, more than 90% of dung beetle species attracted to feces of wild mammals were collected at deer feces [35]. We therefore believed that it was very unlikely that using deer dung would skew the sampling of dung beetle species.

### Collection of animal feces in the wild

In June 2017, we established one 5-km survey route in each of the two study areas (LD and HD) to collect fecal samples. We surveyed the routes once every 2 weeks, twice in each study area. All fresh feces of medium to large mammals found on or along the route were collected. The feces were identified by their smell and shape as from sika deer, Asian black bear, Japanese marten, badger, red fox, masked palm civet, wild boar, and raccoon dog. If we were unable to identify the animal species at the scene, we did not collect the fecal sample. Raccoon dogs and badgers use latrines (fecal pile sites) for defecation. In the surveys, we collected only new fecal samples.

### Stable isotope analysis

Feces obtained from the zoo-housed sika deer and raccoon dogs were used for the breeding experiment as were: exoskeletons (elytra) of the dung beetles that ate these feces during the larval stage in the breeding experiment; dung beetles collected from each study area (species of which at least 10 individuals were collected in each study area); and feces collected in each study area from each wild mammalian species were dried for 72 h at 60°C, powdered, and measured into 0.5-mg aliquots [38].

Each sample was enclosed in a tin cup and combusted in a FlashEA1112 elemental analyzer (Thermo Fisher Scientific, Bremen, Germany) interfaced with a Delta V isotope-ratio mass spectrometer (Thermo Fisher Scientific) to determine the nitrogen isotope ratio.

The results, presented in *δ* notation as parts per thousand (‰) relative to *R*_standard_, were calculated as follows:

$$\delta^{15}N=R_{sample}/R_{standard}-1$$

where *R*_sample_ is the ^15^N*/*^14^N ratio of the sample and *R*_standard_ is the nitrogen isotope ratio of the international standard (atmospheric N_2_). The nitrogen isotope ratios were calibrated against laboratory standards (Shoko Science Co., Ltd., Saitama, Japan), which are traceable back to international standards. The analytical standard deviation (SD) of the stable isotope analysis was ± 0.15‰ for *δ*^15^N.

### Estimating relative mammal abundance and relative amount of feces from each mammal species

We installed automatic cameras (Ltl-Acorn 6210, Ltl Acorn Outdoors, Green Bay, Wisconsin, USA) at 10 locations at 500-m intervals along animal trails in each study area and took still images of mammals from June to September 2017. The cameras were installed two to five meters from the animal trails at heights of 30 to 50 cm. They were checked and the batteries replaced monthly. From these images, we calculated a relative abundance index (RAI: total number of individuals photographed / (number of cameras × number of days as a unit to compare the relative frequencies of images). Because cameras along animal trails do not sample different species at random, the results are biased by such factors as activity patterns and changes in trail use. Thus, they can be used to assess species’ relative abundance between sites, but the data cannot be extrapolated to compare species population densities within defined areas. Thus, we estimated the relative abundance of same mammal species between areas. If the same species was photographed at the same location within 30 min, we discounted the second sighting.

We multiplied the RAI of each animal species and the weight (in grams) of the feces produced by one individual of the species each day in each study area to estimate the relative amount of feces potentially provided by each species in each study area. The mean fecal production (fresh weight) per day per individual of each species during the summer was calculated from mammals in captivity or from data from a previous study (see below). We used adult sika deer (two individuals, fed grass), an adult Japanese macaque (one individual, fed fruit), adult raccoon dogs (four individuals, fed dog food), adult badgers (two individuals, fed dog food), adult masked palm civets (*Paguma larvata*; one individual, fed kiwifruit), and adult martens (two individuals, fed sausage) from the Kanagawa Prefectural Natural Environment Conservation Center for the calculations. We also used adult Asian black bear (two individuals, fed corn and fruit) from the Institute of the Japanese Black Bear in Ani, Akita Prefecture. For wild boars, we assumed a body weight of 60 to 80 kg, but because of a lack of referenced data we used the fecal production value for adult domestic pigs [39]. To compare the relative amounts of feces potentially provided by each species in LD and HD sites, we calculated the relative amount of feces (RAI × mean fecal production (fresh weight) per day per individual of each species during the summer) at both LD and HD sites for each mammal species.

Before we collected our calculations, we submitted our research plan to the Institutional Animal Care and Use Committee of Tokyo University of Agriculture and Technology. However, the committee judged that ethical approval was not required, because we used only feces from zoos or other institutes and did not use living vertebrates. We therefore did not have approval from our committee.

### Statistical analysis

To compare the mean number of each species of dung beetle per pitfall trap in each study area, we used Mann-Whitney’s *U* tests. The nitrogen isotope values (*δ*^15^N values) of the feces (deer and raccoon dog) used in the breeding experiment were compared by using Welch’s *t*-tests. To compare the δ^15^N values of the exoskeletons of dung beetles that, during their larval stage, ate the feces of deer or raccoon dogs during the breeding experiment, we used Student’s *t*-tests. The *δ*^15^N values of feces from each animal species in each study area were compared by using a linear mixed-effect model. We set the *δ*^15^N value of feces as the response variable and the deer density (high or low) as the fixed effect. The probability distribution was defined to follow a normal distribution pattern. We set each mammal species (sika deer, Asian black bear, Japanese marten, Japanese macaque, wild boar, badger, and raccoon dog) as a random effect on the slope to evaluate whether the deer density effect varied among mammal species. This was done because it was possible that changes in deer density could have different effects on the diets of different types of mammal, such as herbivores (sika deer and wild boar) or omnivores (Asian black bear, Japanese marten, Japanese macaque, badger, and raccoon dog). We also set mammal species as a random effect on the intercept to evaluate mammal species–related differences in *δ*^15^N values that were unrelated to deer density. We used the lme4 package [40] to create the models and the MuMIn package [41] to calculate the marginal *R*^2^ to check the degree to which the fixed effect explained the response variable and the conditional *R*^2^ to check the degree to which the fixed effect plus the random effects explained the response variable. We also compared the *δ*^15^N values of the exoskeletons of dung beetles collected in each study area; for this we used Student’s *t*-test, Mann-Whitney’s *U-*test, or Welch’s *t*-test, depending on the variance of the population. For all analyses, we used the statistical analysis software R ver. 3.3.0 [42].

## Results

### Dung beetle fauna

We collected eight species of dung beetle, all tunneller species (no dweller), totaling 335 individuals (LD: seven species and 120 individuals; HD: eight species and 215 individuals: Table 1). The total sampling effort per deer density area was 270 trap nights (3 nights / 9 traps per site × 10 sites per deer density area); the sampling effort was therefore the same in LD and HD. The number of dung beetles caught per trap did not differ significantly between the two study areas, with the exception of *Onthophagus ater*. The number of *O*. *ater* individuals per trap was significantly greater at HD than at LD (Table 1., Z = 2.37, *P* = 0.041).

**Table 1:** Body length (mm) and number of beetles per trap for dung beetles caught in the high- and low-density deer population areas. The sampling effort per trap was three nights.

Species	Body length (mm)	Number of beetles per trap ± SD	
		Deer low density (LD)	Deer high density (HD)
*Phelotrupes auratus*	15.3	1.2 ± 1.1	0.7 ± 0.8
*Phelotrupes laevistriatus*	14.7	1.7 ± 2.4	2.3 ± 2.9
*Liatongus minutus*	9.7	0.1 ± 0.3	0.5 ± 0.7
*Onthophagus fodiens*	9.2	0.5 ± 0.5	1.1 ± 0.8
*Onthophagus lenzii*	8.9	0	0.1 ± 0.3
*Onthophagus ater*	8.2	0.4 ± 0.5	1.0 ± 0.4
*Caccobius jessoensis*	6.4	0.3 ± 0.5	0.2 ± 0.4
*Caccobius nikkoensis*	5.7	0.8 ± 0.9	2.7 ± 2.6

### Stable isotope analysis

**Fig 2:**
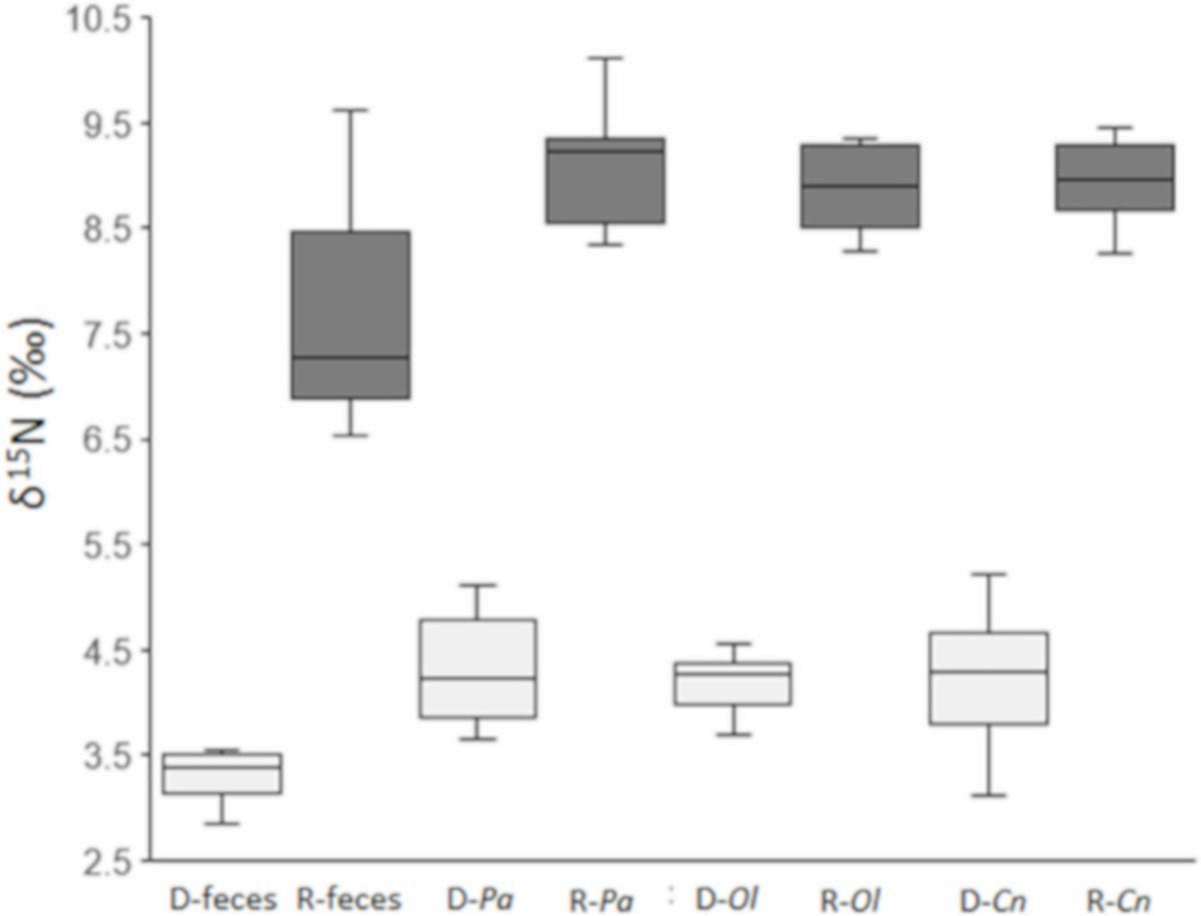
Nitrogen isotope values (*δ*^15^N) of the feces of sika deer, raccoon dogs, and of the exoskeletons of dung beetles. Nitrogen isotope values (*δ*^15^N) of the feces of sika deer (D-feces) or raccoon dogs (R-feces), and of the exoskeletons of dung beetles that fed during their larval stage on each of these types of feces. Dark gray, raccoon dog; light gray, sika deer. D-*Pa* and R-*Pa*, D-*Ol* and R-*Ol*, and D-*Cn* and R-*Cn* are respectively *Phelotrupes auratus*, *Onthophagus lenzii*, and *Caccobius nikkoensis* that fed on sika deer or raccoon dog feces.

Compared with sika deer feces, raccoon dog feces had significantly higher *δ*^15^N values (raccoon dogs (n = 10): 7.6 ‰ ± 0.9 ‰; sika deer (n = 10): 3.4 ‰ ± 0.3 ‰, *t* = –12.70, df = 11.07, *P* = 6.06 x 10^−8^; Fig 2). Among the three species of dung beetle that fed on these feces during their larval stage, those that fed on raccoon dog feces had significantly higher exoskeleton *δ*^15^N values (*P*. *auratus* (n = 7: deer, n = 7: raccoon dog): *t* = –15.63, df = 12, *P* = 2.42 x 10^−9^; *C*. *nikkoensis* (n = 8: deer, n = 8: raccoon dog): *t* = –17.86, df = 14, *P* = 4.95 x 10^−13^; and *O*. *lenzii* (n = 8: deer, n = 8: raccoon dog): *t* = –26.65, df = 14, *P* = 2.13 x 10^−13^: Fig 2).

**Fig 3:**
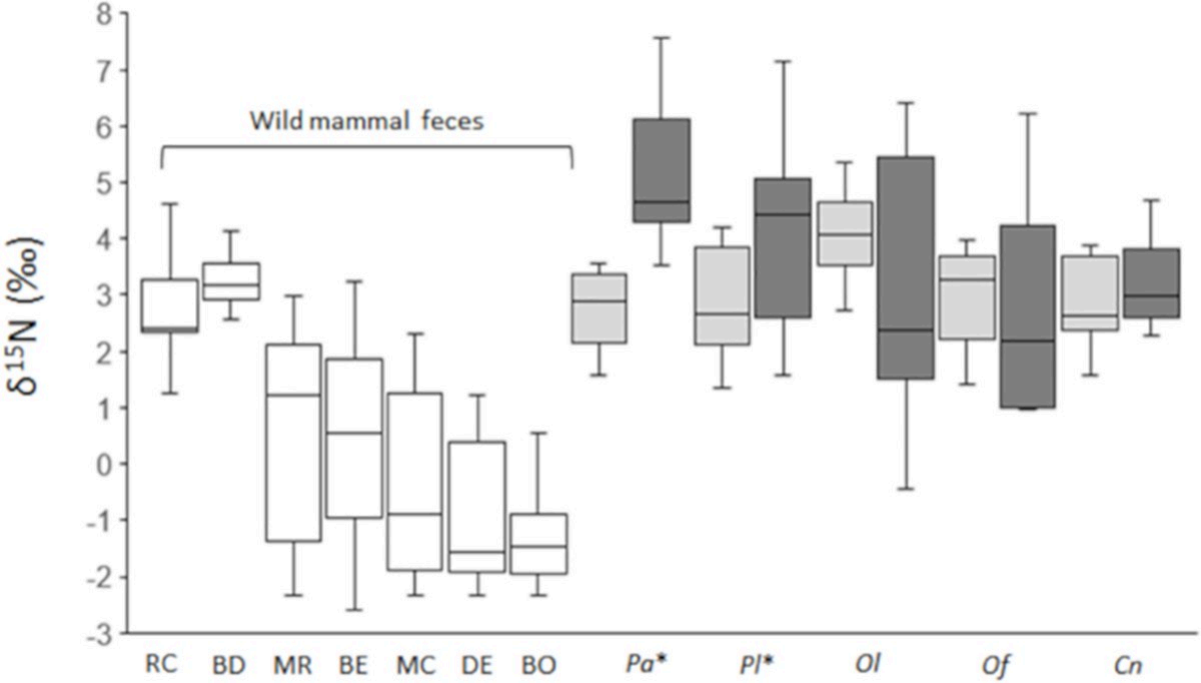
Nitrogen isotope ratios (*δ*^15^N) of each wild mammal species’ feces and of the exoskeletons of dung beetles. Nitrogen isotope ratios (*δ*^15^N) of each wild mammal species’ feces collected in the two study areas (RC: raccoon dog; BD: badger; MR: Japanese marten; BE: Asian black bear; MC: Japanese macaque; DE: sika deer; BO: wild boar) and of the exoskeletons of *Phelotrupes auratus* (*Pa*), *Phelotrupes laevistriatus* (*Pl*), *Onthophagus lenzii* (*Ol*), *Onthophagus fodiens* (*Of*), and *Caccobius nikkoensis* (*Cn*). The ratios in mammal feces did not exhibit significant differences between the two study areas, so the results were pooled. * Indicates species of dung beetles that exhibited significant differences in nitrogen isotope values between the high (light gray) and low (dark gray)-density deer areas.

From among the dung beetles collected in the wild, we measured *δ*^15^N values in five species: *P*. *auratus*, *Phelotrupes laevistriatus*, *Onthophagus fodiens*, *O*. *lenzii*, and *C*. *nikkoensis*. We found that in *P*. *auratus* (HD (n = 6): 2.8 ‰ ± 0.7 ‰; LD (n = 6): 5.1 ‰ ± 1.3 ‰, Student’s *t*-test, *t* = 3.65, df = 10, *P* = 0.004) and *P*. *laevistriatus* (HD (n = 10): 2.9 ‰ ± 0.9 ‰; LD (n = 10): 4.1 ‰ ± 1.6 ‰, Student’s *t*-test, *t* = 2.12, df = 18, *P* = 0.049) the *δ*^15^N values were significantly lower in individuals caught at HD than in those from LD (Fig 3). In the other three species there were no significant differences in the *δ*^15^N values between the two study areas (*O*. *lenzii*: HD (n = 10), 4.1 ‰ ± 0.7 ‰, LD (n = 10), 3.0 ‰ ± 2.1 ‰, Welch’s *t*-test, *t* = –1.44, df = 11.141, *P* = 0.18; *O*. *fodiens*: HD (n = 6), 3.0 ‰ ± 0.8 ‰, LD (n = 5), 2.5 ‰ ± 1.9 ‰, Mann-Whitney’s *U* test, Z = 1.28, *P* = 0.25; *C*. *nikkoensis*: HD (n = 10), 2.8 ‰ ± 0.7 ‰, LD (n = 10), 3.2 ± 0.7 ‰, Student’s *t*-test, *t* = 0.97, df = 18, *P* = 0.35, Fig 3).

**Fig 4:**
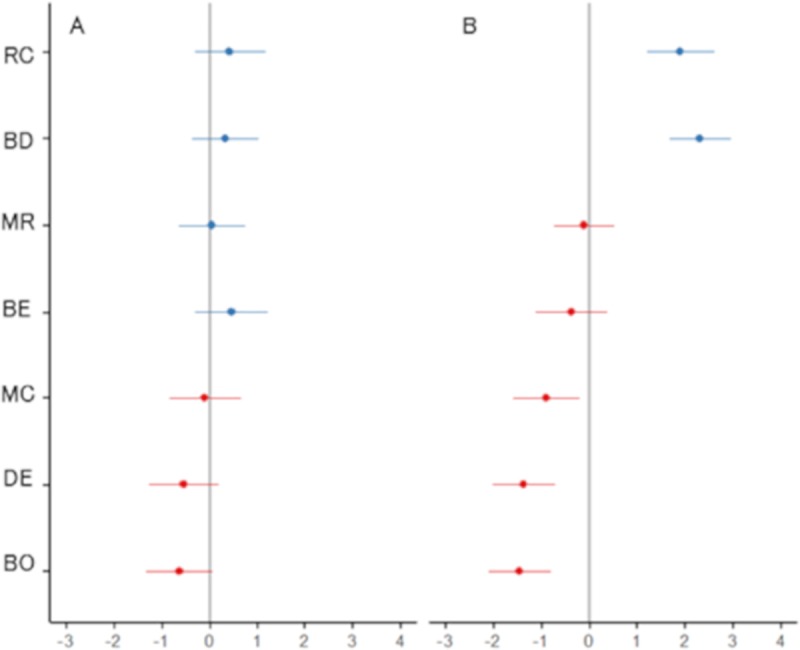
Random effects of each mammal species in a linear mixed-effects model. Error bars show 95% confidence intervals of means. Blue indicates positive mean values and red indicates negative mean values. (A) Random effects on slope, showing the effects of deer density on nitrogen isotope values (*δ*^15^N) in the feces of mammals (RC: raccoon dog; BD: badger; MR: Japanese marten; BE: Asian black bear; MC: Japanese macaque; DE: sika deer; BO: wild boar). (B) Random effects on intercept, showing the *δ*^15^N values in the feces of mammals, unrelated to the effects of deer density.

The *δ*^15^N values of wild mammal feces declined by 0.14‰ on average (*SE* = 0.32, Table 2) when deer density was high. However, deer density did not significantly affect the *δ*^15^N values of wild mammal feces (*P* = 0.69, Table 2). From the marginal *R*^2^, 0.1% of the variance was explained by the fixed effect, and from the conditional *R*^2^, 64.9% of the variance was explained by the fixed effect plus the random effects (Table 2). However, the effect of deer density change depended on the wild mammal species, because the random effect of each mammal species on the slope varied according to the species (Fig 4A). The values of the random effect of sika deer and wild boars on the slope were negative, and the 95% confidence intervals (CIs) of the random effect were predominantly below zero (Fig 4A), indicating that the *δ*^15^N values of the feces of sika deer and wild boars decreased when the deer density increased. For badgers, raccoon dogs, Japanese martens, Japanese macaques, and Asian black bears, the 95% CIs of the random effect on the slope overlapped with zero, indicating that the deer density effect was like the estimated fixed value (Table 2 and Fig 4A). Additionally, the *δ*^15^N values differed depending on the mammal species regardless of deer density, because the random effects on the intercept varied according to the species (Fig 4B). The values of the random effect of badgers and raccoon dogs on the intercept were positive, and the 95% CIs of the random effect were significantly above the 95% CIs of the other species (Fig 4B). The 95% CI of the random effect of Japanese martens on the intercept exceeded the 95% CIs of sika deer and wild boars but overlapped the 95% CIs of Asian black bears and Japanese macaques (Fig 4B); however, these differences were not significant. There were no significant differences in the 95% CI of the random effect on the intercept among Asian black bears, Japanese macaques, sika deer, and wild boars (Fig 4B). Therefore, the *δ*^15^N values of the feces of badgers and raccoon dogs were notably higher than those of the other species, followed by Japanese martens, Asian black bears, and Japanese macaques (consumers of omnivorous plant-based diets), and finally sika deer and wild boars (consumers of plant-based diets). Although badgers and raccoon dogs are omnivores, their summer food habits are heavily biased toward an animal-based diet [43]. We therefore considered them to be consumers of omnivorous animal-based diets.

**Table 2:** Linear mixed-effects model of effect of deer density on nitrogen isotope values (*δ*^15^N) in the feces of wild mammals, with mammal species as a random effect.

Random effect		Variance	SD			
Group: species (n = 7)	Intercept	2.44	1.56			
	Deer density	0.33	0.58			
	Residual	1.68	1.30			
Fixed effect		Coefficient	SE	*t*-value	*P*-value	Marginal R^2^ / Conditional R^2^
Intercept		0.73	0.62	1.19	0.28	0.001 / 0.649
Deer density (high vs. low)		–0.14	0.32	–0.43	0.69	

### Estimation of relative amounts of mammal feces in the study areas

In both study areas, mammal images i.e. sika deer, badgers, raccoon dogs, Japanese martens, masked palm civets, wild boars, Japanese macaques, red foxes, and Asian black bears were captured. Images of sika deer were captured significantly less often at LD (16.4 ± 4.9 times) than at HD (32.1 ± 14.9 times; *P* = 0.001). There were no significant differences in the RAIs of raccoon dogs and badgers between the study areas. In both study areas the RAI of sika deer was the highest (LD: 0.26; HD: 0.57) (Table 3).

**Table 3:** Relative abundance index (RAI) and relative amount of mammal feces (the weight in grams of each species’ feces as a ratio of the total weight of feces from all species) in each study area.

		Sika deer	Raccoon dog	Badger	Japanese marten	Masked palm civet
**RAI**						
	Low deer density (LD)	0.26	0.05	0.03	0.05	0.03
	High deer density (HD)	0.57	0.04	0.02	0.15	0.06
**Relative amount of mammal feces**						
	Low deer density (LD)	115.1	2.2	1.5	0.5	1.0
	High deer density (HD)	250.3 (2.2)	1.8 (0.8)	0.8 (0.6)	1.4 (2.7)	1.7 (1.6)

Values in parentheses are the amounts of each species’ feces as percentages of the amounts in the low-deer-density area.

The mean fecal production (fresh weight) per day per individual of each species during summer was 442.2 ± 34.5 g for sika deer; 15.2 ± 3.5 g for Japanese macaques; 41.2 ± 6.5 g for raccoon dogs; 49.4 ± 5.2 g for badgers; 29.4 ± 7.5 g for masked palm civets; and 9.9 ± 2.5 g for Japanese martens. For Asian black bears, the calculated mean fecal production per day per individual was 467.5 ± 46.8 g. For wild boars, the rate (as determined in domestic pigs) was 2.3 kg.

To estimate the relative amounts of feces between LD and HD for each mammal species, we targeted those mammal species for which the RAI was greater than 0 at both LD and HD; we therefore excluded Japanese macaque (HD: 0.0; LD: 0.2), Asian black bear (HD: 0.0; LD: 0.0), and wild boar (HD: 0.0 LD: 0.0). Only the relative amount of sika deer feces was substantially greater at HD than at LD (HD: 115.1; LD: 250.3; Table 3).

## Discussion

By analyzing the nitrogen isotope ratios of the exoskeletons of adult dung beetles, we could document the usefulness of these ratios in verifying larval diets. Additionally, we found that an increase in sika deer population density may promote a change in the type of feces where adult beetles lay their eggs, altering the diet of dung beetle larvae. Our results confirmed that the above trend was more notable in some larger dung beetle species, supporting our hypothesis. However, further investigation is required to determine the causal mechanism.

It is possible that only large dung beetle species experienced changes in their larval diets because of their preference for larger feces with higher water and nitrogen contents, which they select when laying their eggs [44]. Thus, it is possible that larval diets of large dung beetles changed from the animal-based-diet group to the plant-based diet group. However, we found that the relative amounts of feces of raccoon dogs and badgers did not increase with an increase in deer population density. Thus, other, unknown reasons accounted for the decrease in availability of these feces. Because forests in Japan that are not overpopulated by deer have a dense understory of plants such as *Sasa* spp. [21], the feces excreted by animals in these forests dry relatively slowly, and dung beetles can thus use these feces for relatively long periods of time [25]. Conversely, where deer population density is increasing and understory declining, mammal feces may dry faster, potentially reducing their use by dung beetles. The drying may also reduce the water content of carnivore feces, potentially decreasing the availability of these feces to large dung beetles. However, with an increase in sika deer density, and because deer live in herds, their feces were available in high density for immediate use by the large dung beetles. The feces of sika deer were less nutritious than those of the animal-based diet group, so although the larvae of the large *Phelotrupes auratus* beetles changed their diet, their population did not increase. Contrarily, another large dung beetle species, *P*. *laevistriatus*, tended to increase in population size with increases in deer density. This may have occurred because *P*. *laevistriatus* has a more diverse diet than *P*. *auratus*, utilizing decomposed materials other than animal feces, such as mushrooms [35].

Although *O*. *lenzii* and *O*. *fodiens* did not show significant differences in exoskeleton *δ*^15^N values between the two study areas, variance was greater in the low-deer-density area. For *C*. *nikkoensis* there was no significant difference in *δ*^15^N values or their variation between the two study areas. Additionally, only in one, small species, *Onthophagus lenzii*, did trapping frequency significantly increase with an increase in deer density. Our results are in line with previous studies that showed that an increase in deer density led to an indirect increase in the biomass of small dung beetles [22, 23, 24, 26]. They are relatively small dung beetles, and their recruitment for feces use may not be as narrow as those of the large dung beetle species [44]. Thus, small dung beetles may use the easily obtained feces that is in higher abundance, the deer feces.

We recognize the limitations of our study. First, only tunnellers, no dwellers, were collected. This focal sampling may therefore have been biased toward species that were easier to observe and collect. Second, our breeding experiment had small sample sizes. The natural history of wild dung beetles in Japan remains unknown, so the methods used in our breeding experiment could likely be improved. Future breeding research should consider aspects such as changes in temperature and soil moisture. Third, we do not know the relationship between fitness and diet in large dung beetles. A previous study showed that increases in the deer population did not affect the fecal decomposition function of large dung beetle species [18], but this study did not examine changes in the beetles’ diet. Because our results suggested that large dung beetle species changed the animal species’ feces they used for laying eggs and feeding their larvae, future research needs to examine how changes in the diet affect beetle fitness. If adult dung beetles do indeed switch to deer feces as the deer population increases, then an increase in deer populations may be reducing the beetles’ function in decomposing the feces of animals with animal-based diets, such as raccoon dogs and badgers. The feces of raccoon dogs and badgers have a high nitrogen content and contain the berry tree seeds of many species [45]. On the other hand, deer feces include grass seeds, and most tunneller dung beetles work as secondary dispersers of these seeds [46]. Therefore, an overabundance of sika deer may also affect the diverse ecological functions of dung beetles through a change in their diet, impacting on their material cycle functions and secondary seed dispersal. So, the landscape could become simplified and lose functionality due to an overabundance of sika deer.

## Supporting information

S1 File Raw dataset. The dataset of Table 1, Table 3, Fig 1, and Fig 2.
(XLSX)
